# Development and Validation of an Immune-Related Gene-Pair Model of High-Grade Serous Ovarian Cancer After Platinum-Based Chemotherapy

**DOI:** 10.3389/fonc.2020.626555

**Published:** 2021-02-10

**Authors:** Jiaxing Lin, Xiao Xu, Dan Sun, Tianren Li

**Affiliations:** ^1^ Department of Urology, The First Hospital of China Medical University, Shenyang, China; ^2^ Department of Pediatric Intensive Care Unit, The Shengjing Hospital of China Medical University, Shenyang, China; ^3^ Department of Gynaecology, The First Hospital of China Medical University, Shenyang, China

**Keywords:** high-grade serous ovarian cancer, Cox model, prognostic marker, immune gene, gene pair

## Abstract

**Background:**

High-grade serous ovarian cancer (HGSOC) is a common cause of death from gynecological cancer, with an overall survival rate that has not significantly improved in decades. Reliable bio-markers are needed to identify high-risk HGSOC to assist in the selection and development of treatment options.

**Method:**

The study included ten HGSOC cohorts, which were merged into four separate cohorts including a total of 1,526 samples. We used the relative expression of immune genes to construct the gene-pair matrix, and the least absolute shrinkage and selection operator regression was performed to build the prognosis model using the training set. The prognosis of the model was verified in the training set (363 cases) and three validation sets (of 251, 354, and 558 cases). Finally, the differences in immune cell infiltration and gene enrichment pathways between high and low score groups were identified.

**Results:**

A prognosis model of HGSOC overall survival rate was constructed in the training set, and included data for 35 immune gene-related gene pairs and the regression coefficients. The risk stratification of HGSOC patients was successfully performed using the training set, with a p-value of Kaplan-Meier of < 0.001. A score from this model is an independent prognostic factor of HGSOC, and prognosis was evaluated in different clinical subgroups. This model was also successful for the other three validation sets, and the results of Kaplan-Meier analysis were statistically significant (p < 0.05). The model can also predict patient progression-free survival with HGSOC to reflect tumor growth status. There was a lower infiltration level of M1 macrophages in the high-risk group compared to that in the low-risk group (p < 0.001). Finally, the immune-related pathways were enriched in the low-risk group.

**Conclusion:**

The prognostic model based on immune-related gene pairs developed is a potential prognostic marker for high-grade serous ovarian cancer treated with platinum. The model has robust prognostic ability and wide applicability. More prospective studies will be needed to assess the practical application of this model for precision therapy.

## Introduction

Ovarian cancer is the second most common cause of gynecological cancer death worldwide (1). In 2018, approximately 22,240 new ovarian cancer cases were diagnosed in the United States, and 41,070 patients with ovarian cancer died (2). High-grade serous ovarian cancer (HGSOC) accounts for about 70%–80% of ovarian cancer deaths, and there has been little improvement in overall survival over the decades (3). Many patients suffer relapse and chemotherapy resistance (4) after the standard treatment of HGSOC of optimal debulking surgery and systemic cytotoxic platinum-based chemotherapy. Screening and monitoring of ovarian cancer typically focuses on detection of cancer antigen 125 (CA125) and use of the ovarian cancer risk algorithm (ROMA), but these methods are limited and have poor prognosis ability (5, 6). The Federation International of Gynecology and Obstetrics (FIGO) staging system is used in clinical application, and the correlation between ovarian cancer prognosis and FIGO staging is well confirmed (7). However, the FIGO staging system focuses mainly on clinical features, while ovarian cancer is a disease with high histological and genetic heterogeneity (8). Therefore, there is an urgent need to develop new molecular markers to identify HGSOC subsets with poor survival and high heterogeneity for better selection of clinical treatment options.

Due to advances in bioinformatics, many studies have proposed gene expression signatures for risk stratification in HGSOC patients (9). However, many of these studies utilized excessive fitting or lack sufficient verification, so these models remain far from clinical application (10, 11). There is a significant amount of HGSOC transcriptome data in the public database. However, the heterogeneity between cohorts and the diversity of sequencing platforms have limited use of traditional gene expression data for cross-cohort comparison. Some studies have gene pairs based on ranking of the relative expression levels of two genes, and then construct a prognostic model based on gene pairs. This method eliminates the heterogeneity of different data, and this approach has been successfully verified with reliable results for analysis of many cancers, such as non-small cell lung cancer (12) and colorectal cancer (13). Still, it has not been reported in ovarian cancer.

Ovarian cancer is an immunogenic tumor. The spontaneous anti-tumor immune response of some patients can prolong survival time, while the immune escape of other patients can shorten survival time (14). The main obstacle to implementing immunotherapy for ovarian cancer is an immunosuppressive tumor microenvironment (15). The immune-related biomarkers can predict the response to different types of immunotherapy and promote our understanding of the interactions between molecules and tumor cells in the microenvironment.

In this study, we used multiple transcriptome data sets of HGSOC to develop and verify an individualized prognostic model of HGSOC based on immune-related gene pairs. We used this model to stratify the risk of patients and explore differences in potential immune infiltration and enrichment pathways in high and low-risk patients.

## Materials and Methods

### Study Population and Eligibility Criteria

The search was performed using keywords of “ovarian cancer” and “survival” in the Gene Expression Omnibus (GEO, https://www.ncbi.nlm.nih.gov/geo/) and The Cancer Genome Atlas (TCGA, https://portal.gdc.cancer.gov/) databases. We found a total of 28 cohorts (N = 4,115). First, study cohort screening was carried out. Eighteen cohorts were removed (N = 2,215, **Table S2**) that did not meet the inclusion criteria (treated with platinum; contained HGSOC; contained overall survival data). Ten research cohorts (N = 1900, **Table S1**) were left for further analysis: TCGA-OV (16) from TCGA database, and GSE53963 (17), GSE63885 (18), GSE26712 (19), GSE17260 (20), GSE32062 (21), GSE32063 (21), GSE14764 (22), GSE30161 (23), and GSE9891 (24) from GEO database. Through sample screening, 1,526 HGSOC samples with complete survival data and transcriptional data were found (**Table S3**).

**Figure 1:**
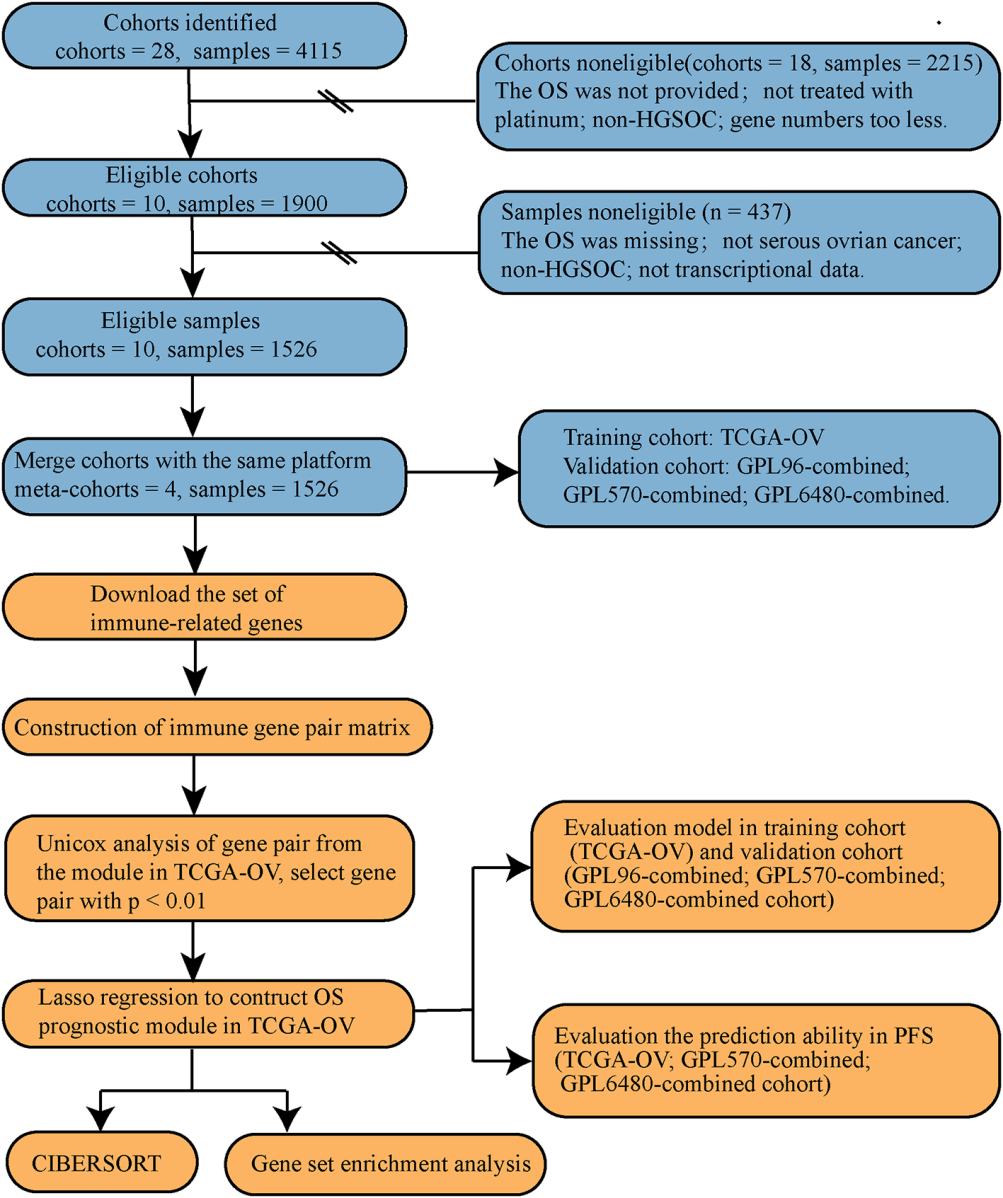
Data processing and research process.

For the specific data processing and research flow, see **Figure 1**.

### Cohort Merging

The research cohorts were merged according to the same microarray platform. There are smaller deviations from cohorts of identical microarray platforms. Merge cohorts can reduce data redundancy and increase data size. We used the combat function of R package “sva” to remove the batch effect. A total of three combined cohorts were merged out of the nine GEO cohorts (**Table 1**). The above three GEO combined cohorts were used as the validation cohort and the TCGA-OV cohort was used as the training cohort.

**Table 1:** The information of combined cohorts.

Dataset-combinde	GEO accession	Platform	Sample size
TCGA-OV	—	Affymetrix Human Genome U133A Array	363
GPL96-combined	GSE14764; GSE26712	Affymetrix Human Genome U133A Array/GPL96	251
GPL570-combined	GSE9891; GSE30161; GSE63885	Affymetrix Human Genome U133 Plus 2.0 Array/GPL570	354
GPL6480-combined	GSE17260; GSE32062; GSE32063; GSE53963	Agilent-014850 Whole Human Genome Microarray 4x44K G4112F (Probe Name version)/GPL6480	558

### Construction of Immune Gene Pair Matrix

An immune gene set was downloaded from Immport (https://www.immport.org/). The immune genes expressed in the TCGA-OV, GPL96-combined, GPL570-combined, and GPL6480-combined cohorts were identified, and the expression value of the genes should be more than 0.5. We defined the combination of two immune-related genes (IRG-1 and IRG-2) as an immune-related gene pair (IRGP). The IGRP score compares the expression levels of IRG-1 and IRG-2 for a given sample. When the expression of IRG-1 is greater than that of IRG-2, the IRGP score is 1; otherwise, the IRGP score is 0. Thus, the immune gene pair matrix is made of 0’s and 1’s. Each IRGP must meet the cohort’s standard that the proportions of 0’s and 1’s must be greater than 20% so that the IRGP is meaningful for subsequent analysis.

### Cox Model Construction

Lasso (Least absolute shrinkage and selection operator) is a statistical method to reduce data dimensionality. Lasso selects the variables of the sample data based on a penalty method. By compressing the original coefficients, insignificant variables whose coefficients become zero are discarded, and any collinear variables are removed. Finally, a simplified model is obtained. We used the R package “glmnet” and “survival” to perform the lasso regression operation (25) and construct the IRGP index (IRGPI) model. First, we used the function “glmnet” for 1,000 random simulations to build the model and obtain the correlation between the coef (regression coefficient) and lambda (punishment coefficient). Then the function “cv.glmnet” is used for random simulation 10-fold cross-validation (CV) 1,000 times. 10-fold CV divides data into ten equal parts. One takes one as a training set and uses other parts to validate models. Deviations obtained by CV can be used to evaluate models, and smaller deviations indicate better models. The model can be expressed as: IRGPI = ∑ n_i_ (IRGP_i_ • coef_i_) (n is the number of IRGP, IRGP_i_ is the score of the ith IRGP, and coef_i_ is the regression coefficient of the ith IRGP).

### Model Verification

We used the Kaplan-Meier method to describe the occurrence of survival outcomes. To do this, we used the R packages “survminer” and “survival” to compare the prognosis for different groups. The function “res.cat” was performed to obtain the best truncation value in continuous variables and then group the samples, requiring each group size to be less than 20% of the total group. Using this method for Kaplan-Meier analysis, the p-value is the smallest. The R package “survival” was then used to perform univariate and multivariate Cox regression analysis to observe the model’s prognosis ability and clinical factors. The R package “survivalROC” was used to draw receiver operating characteristic (ROC) curves and calculate the area under the curve (AUC) values. AUC values greater than 0.5, indicated that the factor can be used as an indicator of prognosis, with values closer to 1 indicating higher accuracy of prognosis.

### Calculation of the Infiltration Level of 22 Kinds of Immune Cells

CIBERSORT (“Cell-type Identification By Estimating Relative Subsets Of RNA Transcripts”) is a complex tissue deconvolution method based on linear support vector regression of gene expression profiles (26). We used the R package “CIBERSORT” and the “Leukocyte signature matrix” to obtain the composition of 22 kinds of immune cells in each sample, and only the samples with p < 0.05 were used for the analysis.

### Gene Set Enrichment Analysis

Gene set enrichment analysis (GSEA) is a computational method used to determine if a predefined set of genes show differences between two biological states (27). According to the risk scores of samples, the samples can be divided into two groups, and the enrichment difference of the two groups can be explored using “c2.cp.kegg.v7.0. Symbols.” P-value < 0.05 and q-value < 0.05 indicate that the enriched items are statistically significant. R packages “ggplot2” and “clusterProfiler” were used to enhance the appearance of the enrichment plot.

### Statistical Analysis

All statistical analysis used in this study is based on R Programming Language software (Rx64 3.5.1). We used the R package “pheatmap” to visualize score distribution, the R package “fmsb” to draw radar maps, and the online website “Sangerbox” (http://sangerbox.com/) to draw Venn diagrams. Wilcoxon test was used to compare between the two groups, and a value of P < 0.05 was considered statistically significant. The online website “Metascape” was used to determine the correlated gene set pathways and perform enrichment analysis (28).

## Results

### Construction of Cox Model Based on IRGP

**Figure 2:**
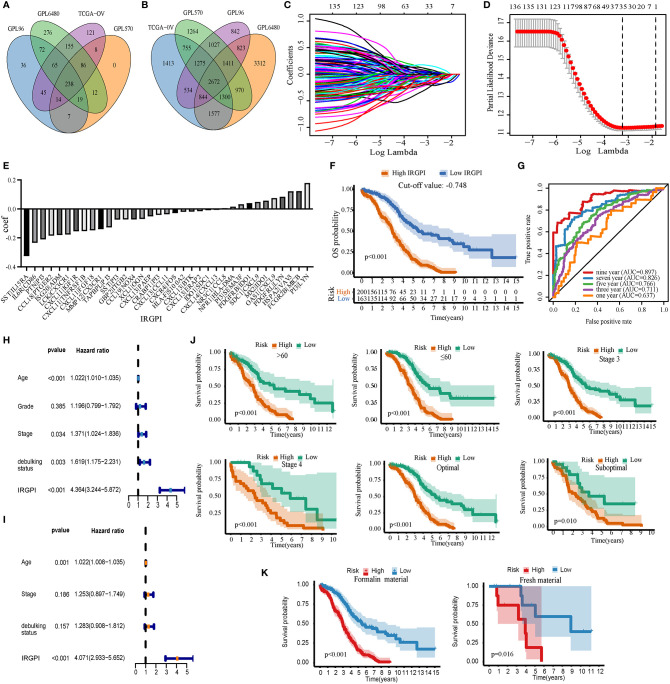
Build and verify immune-related gene pair index (IRGPI) in the training set. **(A)** Venn map of immune genes. **(B)** Venn diagrams of immune gene pairs. **(C)** The diagram of lambda and coef. **(D)** Partial likelihood deviance corresponding to different models. **(E)** The coef value corresponding to each gene. **(F)** IRGPI’s Kaplan-Meier curve of overall survival in the TCGA-OV cohort. **(G)** The ROC curve of IRGPI in TCGA-OV cohort. **(H)** Forest map based on single factor cox analysis. **(I)** Forest map based on multivariate cox analysis. **(J)** Kaplan-Meier analyzed IRGPI under different clinical types. **(K)** Kaplan-Meier analyzed IRGPI under formalin-fixed paraffin-embedded material and fresh material. P < 0.05 indicates that it is statistically significant.

Data for a total of 1526 HGSOC samples that received platinum-based treatment were obtained from the TCGA-OV, GPL96-combined, GPL570-combined, and GPL6480-combined cohorts. TCGA-OV was used as the training set and GPL96-combined, GPL570-combined, and GPL6480-combined cohorts were used as the validation set. We obtained 238 common immune genes from the intersection of the four cohort immune genes (**Figure 2A**). An IRGP analysis was performed by pairwise construction of 238 genes in each cohort. The IRGP that meets the requirements of the four cohorts was then intersected to obtain 2,672 common IRGPs (**Figure 2B**). The relationship between the 2,672 IRGPs and overall survival rates was evaluated using the TCGA-OV cohort, and the results indicated that 141 IRGPs were associated with the prognosis of HGSOC (p < 0.01). The lambda and coef diagrams of IRGPs (**Figure 2C**) were constructed using the lasso algorithm. As the lambda value increases, the coefficients of some IRGPs become zero, which means that the scores of these IRGPs do not affect the model. We then used 10-fold CV to calculate the partial likelihood deviance of the model (**Figure 2D**). The deviance was smallest when 35 genes were used. The coef of each IRGP was obtained according to the corresponding lambda value (**Figure 2E**). The 35 IRGPs and the corresponding coef values constitute the IRGPI prognostic model (**Table S4**). The IRGPI values were calculated for each sample in the four cohorts. These IRGPs include 52 genes. Through pathway and process enrichment analysis, we found that these genes are mainly concentrated in “cell chemotaxis,” “cytokine-mediated signaling pathway,” and “positive regulation of cell migration” (**Table S5**).

### Verification of the Prognostic Ability of IRGPI of Overall Survival Rate in the Training Set

IRGPI significantly stratified HGSOC patients in TCGA-OV, with poor prognosis for high-IRGPI group, and the best cut-off value was −0.748 (p < 0.01, **Figure 2F**). The AUC values were 0.637, 0.711, 0.766, 0.826, and 0.897 for 1, 3, 5, 7, and 9 years, respectively (**Figure 2G**). Univariate Cox analysis showed that age, stage, debulking status, and IRGPI had prognosis ability (p < 0.05, **Figure 2H**). Multivariate Cox analysis showed that age and IRGPI could be used as independent prognostic factors (p < 0.01, **Figure 2I**). IRGPI effectively stratified the risk of HGSOC patients under different clinical types (Age <= 60; age > 60; stage 3; stage 4; optimal; suboptimal) (p < 0.01, **Figure 2J**). IRGPI also had good prognosis ability bath in formalin-fixed paraffin-embedded material and fresh material (p < 0.01, **Figure 2K**). Overall, IRGPI can be used to independently evaluate the overall survival rate of patients with HGSOC.

### Prognostic Ability of the Overall Survival Rate of IRGPI in the Validation Sets

**Figure 3:**
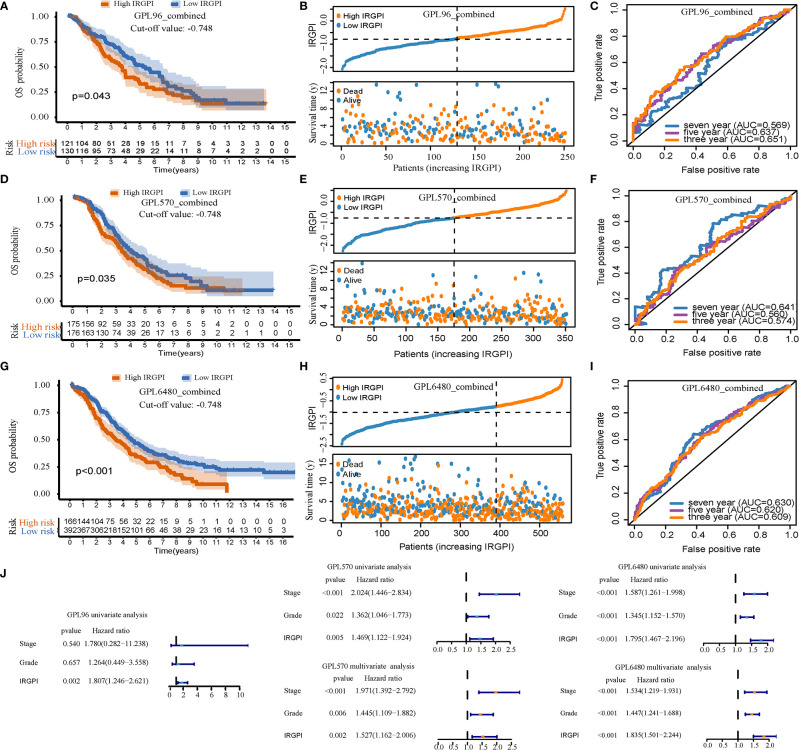
The prognostic ability of immune-related gene pair index (IRGPI) was verified using the verification set. **(A–C)** For the GPL96-combined cohort, the Kaplan-Meier curve, patient distribution, and ROC curve of IRGPI. **(D–F)** For the GPL570-combined cohort, the Kaplan-Meier curve, patient distribution, and ROC curve of IRGPI. **(G–I)** For the GPL6480-combined cohort, the Kaplan-Meier curve, patient distribution, and ROC curve of IRGPI. P < 0.05 indicates that it is statistically significant. **(J)** Univariate and multivariate analysis of three cohorts.

IRGPI can be used as a prognostic factor for the GPL96-combined, GPL570-combined, and GPL6480-combined cohorts. After calculating the IRGPI of the three validation sets, we used the cut-off value (−0.748) of the TCGA-OV cohort to divide the sample into high-IRGPI and low-IRGPI groups. In the GPL96-combined cohort, the results of Kaplan-Meier analysis showed worse prognosis of patients with the high-IRGPI group compared to the low-IRGPI group (p = 0.043, **Figure 3A**); The IRGPI distribution and patient survival status plots are shown in **Figure 3B**; The AUC values for 3, 5, and 7 years were 0.651, 0.637, and 0.569, for the GPL96-combined, GPL570-combined, and GPL6480-combined cohorts, respectively (**Figure 3C**). Similarly, in the GPL570-combined cohort, Kaplan-Meier analysis showed worse prognosis of patients for the high-IRGPI group compared to that of the low-IRGPI group (p = 0.035, **Figure 3D**); **Figure 3E** shows that IRGPI distribution and patient survival status plots; and the AUC values for 3, 5, and 7 years were 0.574, 0.560, and 0.641, respectively (**Figure 3F**). Finally, in the GPL6480-combined cohort, Kaplan-Meier analysis showed worse prognosis of patients for the high-IRGPI group compared to the low-IRGPI group (p < 0.001, **Figure 3G**). **Figure 3H** shows the IRGPI distribution and patient survival status plots, and the AUC values for the three, five, and seven years were 0.609, 0.620, and 0.630, respectively (**Figure 3I**). Univariate results showed that IRGPI was statistically significant in all three verification cohorts (p < 0.05, **Figure 3J**). In the multivariate analysis of GPL570-combined and GPL6480-combined cohorts, IRGPI can analyze the prognosis independently of stage and grade (p < 0.05, **Figure 3J**). Overall, IRGPI successfully stratified the risk of HGSOC patients for the three validation sets.

### IRGPI Can Also Predict Progression-Free Survival (PFS) Probability in Patients With HGSOC

**Figure 4:**
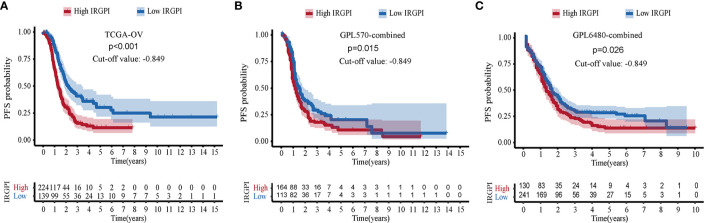
Verification of immune-related gene pair index (IRGPI)’s ability to predict PFS. **(A)** Kaplan-Meier curve of PFS in TCGA-OV cohort. **(B)** GPL570-combined cohort. **(C)** GPL6480-combined cohort.

Including only samples with PFS information, 1011 cases of HGSOC were further analyzed, including samples from the TCGA-OV (N = 363), GPL570-combined (N = 277), and GPL6480-combined cohorts (N = 371). Kaplan-Meier analysis showed that IRGPI allowed risk stratification of HGSOC in TCGA-OV, and the best cut-off value was −0.849 (p < 0.001, **Figure 4A**). With the value −0.849, the GPL570-combined was separated into two groups. Kaplan-Meier analysis showed it was significant (p = 0.015, **Figure 4B**). Finally, in the GPL6480-combined cohort, it was found that HGSOC can still predict PFS (p = 0.026, **Figure 4C**). Low PFS correlated with high IRGPI.

### High-IRGPI Group Showed Low Macrophages M1 Infiltration

**Figure 5:**
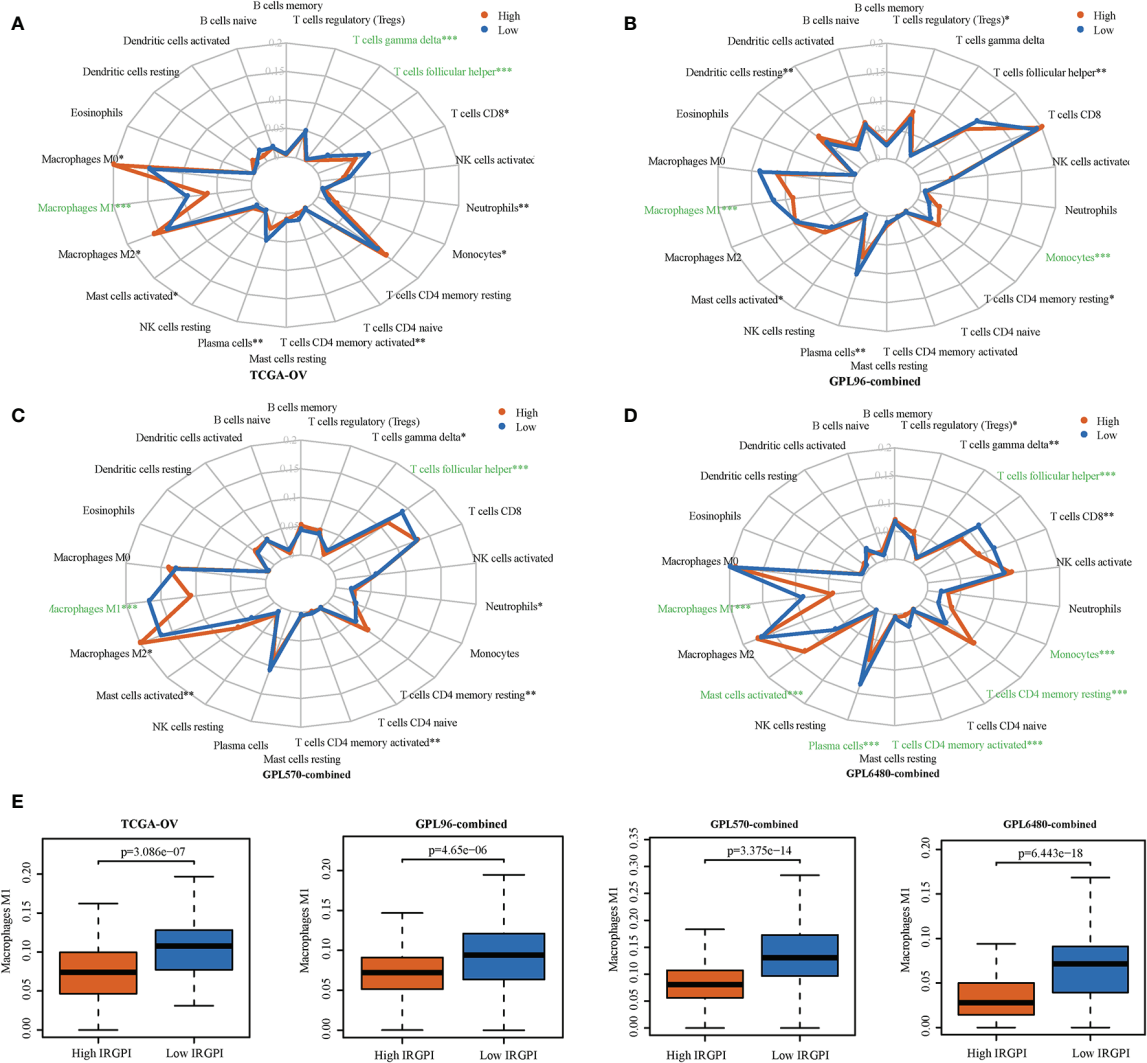
The infiltration levels of immune cells were compared for the high-immune-related gene pair index (IRGPI) and the low-IRGPI groups. **(A)** Infiltration level radar map of TCGA-OV cohort. **(B)** GPL96-combined cohort. **(C)** GPL570-combined cohort. **(D)** GPL6480-combined cohort. **(E)** A difference in the infiltration of M1 macrophages M1 was observed between the two groups. P < 0.05 indicates that this difference is statistically significant. *p < 0.05, **p < 0.01, ***p < 0.001.

We analyzed the differences in the infiltration levels of immune cells between the high-IRGPI and low-IRGPI groups. The radar map presented in **Figure 5A** shows a significant difference in the level of infiltration in the TCGA-OV cohort for M1 macrophages, gamma delta T cells, and follicular helper T cells (p < 0.001). In the GPL96-combined cohort, significant differences were seen for M1 macrophages and monocytes (p < 0.001, **Figure 5B**); in the GPL570-combined cohort, differences were seen for M1 macrophages and follicular helper T cells (p < 0.001, **Figure 5C**); in the GPL6480-combined cohort, differences were seen for M1 macrophages, activated mast cells, plasma cells, monocytes, CD4 memory activated T cells, CD4 memory resting T cells, and follicular helper T cells (p < 0.001, **Figure 5D**). Among these immune cells, only M1 macrophages showed significant differences in high-IRGPI and low-IRGPI groups for all four cohorts. M1 macrophages exhibited higher infiltration level in the low-IRGPI group than that in the high-IRGPI group (p < 0.001, **Figure 5E**).

### Gene Set Enrichment Analysis Based on IRGPI

**Figure 6:**
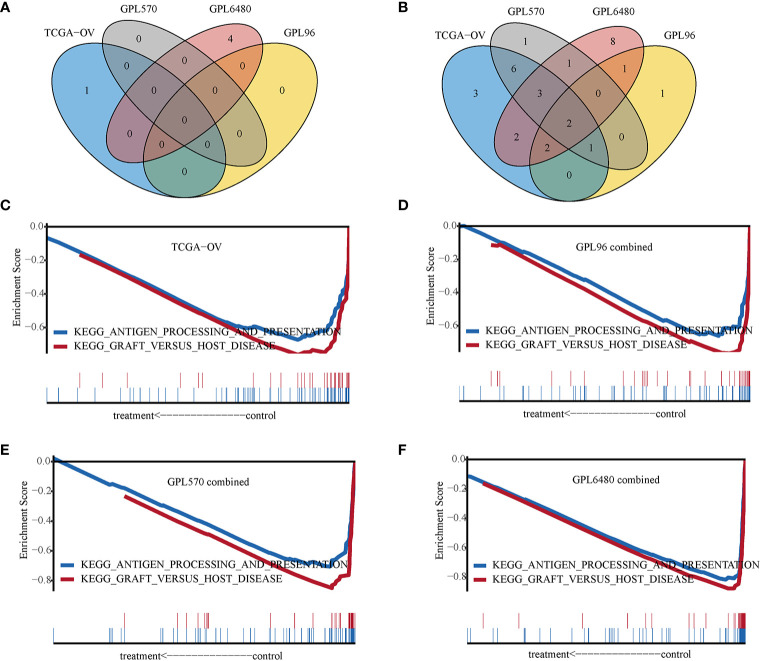
Gene set enrichment analysis. **(A)** Venn diagram of High-immune-related gene pair index (IRGPI) group enrichment pathways. **(B)** Venn diagram of Low-IRGPI group enrichment pathways. **(C)** GSEA line chart of the KEGG “antigen processing and presentation” and “graft versus host disease” pathway in TCGA-OV cohort. **(D)** GPL96-combined cohort. **(E)** GPL570-combined cohort. **(F)** GPL6480-combined cohort.

IRGPI was applied to divide the samples into high-IRGPI and low-IRGPI groups to analyze differences in the enriched GSEA KEGG pathways between the two groups. The results showed few enrichment pathways in the high-IRGPI group, and no pathway is enriched in four cohorts at the same time (**Figure 6A**). In contrast, in the low-IRGPI group, many pathways were enriched, and two pathways were enriched in all four cohorts (**Figure 6B**). The two pathways were “antigen processing and presentation” and “graft versus host disease.” The enrichment plots presented in **Figures 6C–F** show the profile of the running enrichment score and gene-set members’ positions on the rank-ordered list. This result suggests that the better prognosis of the low-IRGPI patients is related to this immune-related pathway’s activity.

## Discussion

Epithelial ovarian cancer is usually detected in advanced stages and has a poor prognosis, making it the second common cause of death from gynecological cancer (29). HGSOC is complex, with varying biological and molecular characteristics, so the realization of personalized precision medicine is the biggest challenge for treatment of HGSOC (1). Reliable prognostic bio-markers are needed to stratify the risk of HGSOC patients to facilitate decisions about treatment options such as chemotherapy, targeted drugs, or immunotherapy. In this study, we screened data from HGSOC patients treated with platinum therapy. We developed an overall survival and prognostic model of HGSOC based on 35 immune-related gene pairs. The model exhibited good prognosis ability for four integrated data sets (n = 1,526). We also detected an obvious activity of M1 macrophages and enrichment of the “antigen processing and presentation” and “graft versus host disease” KEGG pathway in the low-risk group.

The IRGPI model was constructed using 35 IRGPs, a model that can stratify HGSOC patient risk based on platinum therapy. We did a comprehensive search of HGSOC transcriptome data from the TCGA and GEO databases. We included a total of 10 cohorts that met the requirements (n = 1,526), thus this analysis of HGSOC patients is the largest (highest number of samples) to date, increasing the reliability of the conclusions. To prevent data redundancy, we merged the cohorts into four datasets. Most traditional prognostic models are based on gene expression, but this method is unstable for application to a cross-cohort platform. To prevent the technical deviation of genes due to different experimental platforms, we used the relative expression of genes as a unit. This method compares different genes in a single sample, so there is no need to standardize the data. This characteristic would facilitate the application of IRGPI to practical clinical use. The TCGA-OV cohort was used to construct IRGPI, and the cut-off value of the IRGPI score was −0.748. The OS of patients was estimated successfully in four cohorts by using the IRGPI model. The results show that the IRGPI model has robust prognosis ability and wide adaptability. Additionally, the IRGPI model can estimate the PFS of patients with HGSOC (n = 1,011, cut-off value = −0.849) and individually predict disease progression.

Many tumor immune-related biomarkers have been developed, which are potential immune targets that can help guide the proper treatment of patients. This study is the first comprehensive analysis of the immune gene prognosis of HGSOC. Most genes used to construct gene pairs are cytokines and cytokine receptors, which act in cell chemotaxis, angiogenesis, and tumor escape (30, 31). We compared the infiltration level of immune cells between the high-IRGPI and low-IRGPI groups. M1 macrophages showed the most obvious difference, with obvious infiltration inhibition in the high-IRGPI group. Macrophages are important immune cells involved in inflammation and tumorigenesis (32). Macrophages that infiltrate around tumor cells are called tumor-associated macrophages (TAM). TAM include anti-tumor M1 macrophages and tumor-promoting M2 macrophages (33). Studies have shown that a high M1/M2 ratio in ovarian tumor tissue is associated with prolonged survival (34). M2 macrophages can release immunosuppressive factors to support immune escape of ovarian cancer (35). Paclitaxel is used to treat ovarian cancer by polarizing M2 into M1 macrophages in a TLR4-dependent manner (36). Our study shows significant inhibition of M1 macrophages in high-risk patients, suggesting that paclitaxel treatment for HGSOC in high-IRGPI may improve patient survival. In the enrichment analysis, the low-IRGPI group showed enrichment of immune-related pathways, indicating that when the immune pathway is active, the risk of HGSOC is low. The high-IRGPI group may experience immunosuppression, so the prognosis is poor. To summarize, the imbalance of immune function may explain the difference in survival among the patient groups defined by IRGPI.

Although we have used multiple HGSOC cohorts to verify our model with good results, there are limitations of our conclusions. This study is based on large-scale cohorts in a network database and the findings were not verified by additional data. We used the relative expression of genes in a single sample to build the model. Although this method can effectively reduce the sample’s batch processing effect, there may be some complex internal and external interference factors that affect the results. The functions of the genes that make up the identified gene pair with high prognostic ability have not been investigated. Overall, more biological experiments are needed to verify the functional mechanism of ovarian cancer.

## Conclusions

The immune gene pair-based model developed in this study is a good prognostic indicator for high-grade serous ovarian cancer. This model is suitable for individualized selection of medical treatment options to improve patient survival time and quality. Additionally, prospective studies are needed to verify the accuracy of the model and evaluate the clinical efficacy of the model for individualized treatment.

## Data Availability Statement

Publicly available datasets were analyzed in this study. These data can be found here: The TCGA-OV data set can be gained from TCGA database (https://cancergenome.nih.gov/). Nine GEO data sets (GSE53963, GSE63885, GSE26712, GSE17260, GSE32062, GSE32063, GSE14764, GSE30161, and GSE9891) can be gained from GEO database (https://www.ncbi.nlm.nih.gov/geo/).

## Author Contributions

TL provided ideas and solutions. JL and XX for data analysis and drafted preparation. DS downloaded and preprocessed data. All authors contributed to the article and approved the submitted version.

## Conflict of Interest

The authors declare that the research was conducted in the absence of any commercial or financial relationships that could be construed as a potential conflict of interest.

## Abbreviations

HGSOC, high-grade serous ovarian cancer; TCGA, The Cancer Genome Atlas; GEO, gene expression omnibus; AUC, area under the curve; ROC, receiver operating characteristic; IRGP, immune-related gene pair; IRGPI, IRGP index; Lasso, least absolute shrinkage and selection operator; coef, regression coefficient; GSEA, gene set enrichment analysis; PFS, progression-free survival; OS, overall survival.
